# Suicide Attempt in a 38-Year-Old Patient on Varenicline While Intoxicated With Alcohol

**DOI:** 10.7759/cureus.10415

**Published:** 2020-09-12

**Authors:** Ismet Yesilada, Edward Bach, Luba Leontieva

**Affiliations:** 1 Psychiatry, State University of New York Upstate Medical University, Syracuse, USA

**Keywords:** varenicline, alcohol, chantix, suicide, suicidal behavior, intoxication, amnesia, blackout, smoking, smoking cessation

## Abstract

Varenicline is one of two non-nicotine replacement therapy medications approved by the FDA for smoking cessation. It has also demonstrated some success in trials for the treatment of alcohol use disorder. In this case report, we present a case of a 38-year-old male with a history of alcohol and tobacco use disorders and no other psychiatric history, including no history of suicidal ideation or suicide attempts, who was admitted to an inpatient psychiatric facility after a suicide attempt while acutely intoxicated with alcohol one week after starting varenicline treatment for smoking cessation. Reports from the media, and literature from the FDA and manufacturers of CHANTIX^®^ have mentioned a potential interaction between varenicline and alcohol that can subsequently cause "unusual and aggressive behaviors" that are "often accompanied by amnesia to the events"; however, we were unable to find any case reports related to a potential interaction between the two causing a suicide attempt in a PubMed search. Therefore, to our knowledge, this represents the first case report of its kind.

## Introduction

Varenicline is a medication prescribed for smoking cessation. Along with bupropion, it is one of the two FDA-approved non-nicotine replacement therapy medications in the United States [1]. It works by partially activating the α4β2 nicotinic acetylcholine receptor subtype, for which it has a stronger binding affinity than nicotine but weaker efficacy [2]. During nicotine withdrawal, upon binding to this receptor, varenicline stimulates the release of dopamine from the ventral tegmental area into the nucleus accumbens, albeit at a significantly lower rate than nicotine, thus alleviating the negative symptoms and cravings [2]. Varenicline also prevents nicotine from binding to these receptors, thus decreasing the rewarding and reinforcing effects of smoking [3]. In this aspect, varenicline’s mechanism of action is similar to that of buprenorphine in its role in opioid withdrawal treatment [4]. Varenicline, independent of its use in smoking cessation, has also shown efficacy in treating alcohol use disorder by reducing cravings [5-7].

A black box warning mandated by the FDA came in 2009 after a series of reports concerning neuropsychiatric events, including depression, psychosis, and homicidal and suicidal ideation and behavior, with some of these events found to be worsened by alcohol [8-10]. Conflicting data thereafter lead to the Evaluating Adverse Events in a Global Smoking Cessation Study (EAGLES) [11], which investigated the “relative neuropsychiatric safety risk and efficacy of varenicline and bupropion with nicotine patch and placebo in smokers with and without psychiatric disorders”. The data from this double-blind, triple-dummy, placebo- and active-controlled study “did not show a significant increase in neuropsychiatric adverse events attributable to varenicline or bupropion relative to nicotine patch or placebo”, but did demonstrate that “varenicline was more effective than placebo, nicotine patch, and bupropion in helping smokers achieve abstinence”. The study funded by Pfizer (makers of CHANTIX®) and GlaxoSmithKline (makers of Zyban® [bupropion hydrochloride]) led the way of the FDA removing the black box warning [12]. Prior to the EAGLES study being completed in 2015, the FDA released a warning and updated the label for CHANTIX related to “potential alcohol interaction” among other concerns [13]. Furthermore, the Highlights of Prescribing Information by Pfizer, an online prescribing guide for physicians, reports “increased effects of alcohol” and recommends instructing patients to reduce their alcohol intake while taking CHANTIX [14].

## Case presentation

A 38-year-old Caucasian male with a history of severe alcohol and tobacco use disorder and no other prior psychiatric or medical history was admitted as a transfer from an outside hospital’s emergency department to our inpatient psychiatry unit after a suicide attempt by hanging while under the influence of alcohol. He had spent his day at a company party and then spent time with family before going to his garage to do mechanical work, as he regularly does. Throughout the day, he had consumed a significant amount of alcohol, which he suspects was about 18 beers, slightly more than his typical 10-15 beers daily, though he reported that drinking as much as 18 beers was not unusual for him. While working in his garage that night, he sent his significant other concerning text messages and she went to check on him. She found him cyanotic in the face while hanging with a rope around his neck. She likely saved his life as she removed the rope from his neck and called emergency medical services who brought him to our hospital. The patient reported being amnestic to these events and only being aware of them as his significant other had reported them to him.

Throughout the patient’s hospitalization, he was pleasant and cooperative with care. A thorough psychiatric review of systems did not reveal any conditions, except for alcohol use disorder (severe) and tobacco use disorder (severe). He said that he has been using both substances for more than 20 years. Collateral information was obtained from the patient's fiancée, who said that he has been drinking daily for some time. He continually denied any ability to recall the suicide attempt and was distraught regarding the event, especially related to the thought of leaving his significant other and two children without their partner and father, respectively. In conversations, he did admit to increased stress at work due to a promotion many months before, which he attributed to an increase in the amount of alcohol he was drinking as well as increased stress due to having an infant daughter. He noted that the amount of alcohol he had consumed was greater than he typically had been drinking but that he had not previously had a “blackout” with this amount of alcohol. Another possibility considered was amnesia secondary to traumatic asphyxiation or head injury; however, the patient did not demonstrate any cognitive or functional deficits during his inpatient hospitalization nor any signs of this on physical examination. The patient's laboratory value for ethanol was 0.182, and his urine drug screen was negative. He denied any history of illicit and prescription drug abuse.

During the course of evaluation, he noted that he had begun varenicline for cigarette cessation one week ago. He had twice previously used varenicline for the cessation of cigarettes in the past, but both of those times he had also quit alcohol simultaneously. He had relapsed on both occasions, and therefore this time he decided to try quitting only cigarettes with varenicline. In our review of varenicline’s literature, it was notable that what had occurred to our patient was similar to the warning on the prescriber’s information packet, namely unusual and/or aggressive behavior (in this case, toward the self) and amnesia related to the event.

During his stay, the patient was started on the Clinical Institute Withdrawal Assessment for Alcohol (CIWA) protocol, but consistently scored 0 during all assessments. He was started on disulfiram and nicotine replacement therapy while hospitalized, which were both well-tolerated. His hospitalization was otherwise uneventful, and he was discharged three days later.

## Discussion

Varenicline, as one of two medications approved by the FDA for smoking cessation, represents an important treatment for many people. Its safety and efficacy have been demonstrated and its side effects have been described. It has also been the subject of thorough investigation regarding its neuropsychiatric adverse effects. In 2009, a black box warning was added to the medication, which was rescinded in 2016 following the EAGLES trial. Prescribing patterns were significantly altered following these decisions, with a sharp decline in prescriptions following the black box warning, which was then followed by a sharp increase in usage after the repeal of the warning [15].

A PubMed search, using the parameters "varenicline", "suicide", and "alcohol" without any filters, did not return any other case reports. The suicide attempt described in this case report appears to represent the first of its kind and an important example of the risk, which Pfizer, under the guidance of the FDA, publishes on the prescribing package for CHANTIX: “There have been postmarketing reports of patients experiencing increased intoxicating effects of alcohol while taking CHANTIX. Some cases described unusual and sometimes aggressive behavior, and were often accompanied by amnesia for the events. Advise patients to reduce the amount of alcohol they consume while taking CHANTIX until they know whether CHANTIX affects their tolerance for alcohol”. It might also be advisable to consider abstinence if clinically appropriate.

Given the amount of co-morbid alcohol and tobacco use, the frequency of prescription of varenicline, and the ongoing studying regarding the potential viability of varenicline as an option for alcohol use disorder, the authors of this study consider the knowledge of cases like these can be crucial in advancing clinical knowledge regarding the prescription of varenicline. Specifically, we recommend that a thorough review of alcohol consumption history with validated measures, such as the Timeline Followback method [16], and collateral report from family members on alcohol use be taken prior to initiating varenicline.

## Conclusions

Varenicline is an important medication for many people that aids in tobacco cessation. Following its approval in 2006, an FDA black box warning was added in 2009 related to neuropsychiatric adverse effects including suicidality. Following investigation into its efficacy and adverse effects, the black box warning was rescinded in 2016. In the course of its investigation of the neuropsychiatric adverse effects, the FDA added a warning in 2015 related to alcohol use while taking varenicline, and currently Pfizer recommends prescribers to “[instruct] patients to reduce the amount of alcohol they consume until they know whether CHANTIX affects them". Our patient with no prior suicide attempts continued alcohol use (and slightly increased it on the day of his suicide attempt), resulting in a rare “blackout” for him and a subsequent suicide attempt. This occurred one week after the initiation of varenicline. This suicide attempt appears to represent an example of the behavior about which the FDA and Pfizer warn. This case report then represents the first of its kind related to a previously known side effect and the risk of alcohol consumption, and the recommendation by the manufacturer should be more strongly emphasized in conversations between physicians and patients.

## Appendices

**Table 1:** Manufacturer's warnings on possible adverse neuropsychiatric events with drinking alcohol while taking Chantix

Warnings
Increased intoxicating effects of alcohol
Unusual and aggressive behavior
Accompanying amnesia for the events

## References

[1] Jiloha RC (2014). Pharmacotherapy of smoking cessation. Indian J Psychiatry.

[2] Singh D, Saadabadi A (2020). Varenicline. StatPearls [Internet].

[3] LeSage MG, Shelley D, Ross JT, Carroll FI, Corrigall WA (2009). Effects of the nicotinic receptor partial agonists varenicline and cytisine on the discriminative stimulus effects of nicotine in rats. Pharmacol Biochem Behav.

[4] Lutfy K, Cowan A (2004). Buprenorphine: a unique drug with complex pharmacology. Curr Neuropharmacol.

[5] Litten RZ, Ryan ML, Fertig JB (2013). A double-blind, placebo-controlled trial assessing the efficacy of varenicline tartrate for alcohol dependence. J Addict Med.

[6] McKee SA, Harrison EL, O'Malley SS (2009). Varenicline reduces alcohol self-administration in heavy-drinking smokers. Biol Psychiatry.

[7] Mitchell JM, Teague CH, Kayser AS, Bartlett SE, Fields HL (2012). Varenicline decreases alcohol consumption in heavy-drinking smokers. Psychopharmacology.

[8] (2020). Public Health Advisory: FDA Requires New Boxed Warnings for the Smoking Cessation Drugs Chantix and Zyban. http://wayback.archive-it.org/7993/20170112005513/http://www.fda.gov/Drugs/DrugSafety/PostmarketDrugSafetyInformationforPatientsandProviders/ucm169988.htm.

[9] Cinemre B, Akdag ST, Metin O, Doganavsargil O (2010). Varenicline-induced psychosis. CNS Spectr.

[10] Kaplan K., 2020 2020 (2020). Quitting smoking? FDA flags alcohol, seizure risks for Chantix users. https://www.latimes.com/science/sciencenow/la-sci-sn-chantix-alcohol-drinking-seizures-20150310-story.html.

[11] Anthenelli RM, Benowitz NL, West R (2016). Neuropsychiatric safety and efficacy of varenicline, bupropion, and nicotine patch in smokers with and without psychiatric disorders (EAGLES): a double-blind, randomised, placebo-controlled clinical trial. Lancet.

[12] (2020). FDA Drug Safety Communication: FDA revises description of mental health side effects of the stop-smoking medicines Chantix (varenicline) and Zyban (bupropion) to reflect clinical trial findings. https://www.fda.gov/drugs/drug-safety-and-availability/fda-drug-safety-communication-fda-revises-description-mental-health-side-effects-stop-smoking.

[13] Center for Drug Evaluation and Research. (2015 (2020). FDA Drug Safety Communication: FDA updates label for stop smoking drug Chantix (varenicline) to include potential alcohol interaction, rare risk of seizures, and studies of side effects on mood, behavior, or thinking. https://www.fda.gov/drugs/drug-safety-and-availability/fda-drug-safety-communication-fda-updates-label-stop-smoking-drug-chantix-varenicline-include.

[14] (2020). Highlights of Prescribing Information. https://labeling.pfizer.com/ShowLabeling.aspx?id=557.

[15] Desai RJ, Good MM, San-Juan-Rodriguez A, Henriksen A, Cunningham F, Hernandez I, Good CB (2019). Varenicline and nicotine replacement use associated with US Food and Drug Administration drug safety communications. JAMA Netw Open.

[16] Agrawal S, Sobell M, Sobell L (2009). The timeline followback: a scientifically and clinically useful tool for assessing substance use. Calendar and Time Diary.

